# Development and Application of an ELISA Assay Using Excretion/Secretion Proteins from Epimastigote Forms of *T. cruzi* (ESEA Antigens) for the Diagnosis of Chagas Disease

**DOI:** 10.1155/2012/875909

**Published:** 2012-09-25

**Authors:** Mariolga Berrizbeitia, Milagros Figueroa, Brian J. Ward, Jessicca Rodríguez, Alicia Jorquera, Maria A. Figuera, Leomerys Romero, Momar Ndao

**Affiliations:** ^1^Laboratorio de Diagnóstico Serológico en Enfermedades Infecciosas, Postgrado en Biología Aplicada, Universidad de Oriente, Núcleo de Sucre, Cumana 6101, Venezuela; ^2^Instituto de Biomedicina y Ciencias Aplicadas, Universidad de Oriente, Núcleo de Sucre, Cumana 6101, Venezuela; ^3^National Reference Centre for Parasitology, Research Institute of the McGill University Health Centre, Montreal General Hospital, 1650 Cedar Avenue R3-137, Montreal, QC, Canada H3G 1A4; ^4^Centro de Investigaciones en Ciencias de la Salud, de la Universidad de Oriente, Núcleo de Anzoátegui, Barcelona 6001, Venezuela; ^5^Departamento de Bioanálsis, Universidad de Oriente, Núcleo de Sucre, Cumana 6101, Venezuela

## Abstract

An indirect enzyme-linked immunoabsorbent assay (ELISA) for *Trypanosoma cruzi* was developed using epimastigote secretion/excretion proteins (ESEA antigens) obtained from axenic culture supernatants. A panel of 120 serum samples from subjects with confirmed Chagas disease (*n* = 50), healthy controls (*n* = 50), and patients with other parasitic diseases (*n* = 20) was used to evaluate the new ESEA-based ELISA (ELISA_ESEA_). This new test had excellent sensitivity (98%) and acceptable specificity (88%). Cross-reactivity was observed largely in sera from subjects with *Leishmania* and *Ascaris* infections. Using Western blotting and epimastigotes from two distinct *T. cruzi* isolates, several polypeptide bands with molecular masses ranging from 50 to 220 kDa were detected in pooled chagasic sera. However, the band pattern for each isolate was different. These data suggest that an inexpensive and technically simple ELISA based on ESEA antigens is a promising new tool for the diagnosis of Chagas disease.

## 1. Introduction

Chagas disease, discovered by Dr. Carlos Chagas in 1909, is caused by the protozoan parasite, *Trypanosoma cruzi. *This parasite can be transmitted by triatomine vectors, blood transfusions, laboratory accidents, organ transplantation, or through vertical transmission. The disease is endemic in 18 Latin American countries. In 2008, the World Health Organization (WHO) estimates that ~8 million people were infected resulting in ~11 000 deaths per year [1].

Despite decades of effort, there is still no gold standard test for the diagnosis of Chagas disease and several international organizations have recently emphasized the need for improved serodiagnostic tests [1]. To this end, many companies and research groups have developed a range of parasitologic, serologic, and nucleic acid-based assays. The most common methods include the indirect immunofluorescence assay (IFA), indirect haemagglutination assay (IHA), and the enzyme-linked immunosorbent assay (ELISA) [2]. The target antigens in these latter assays have included whole fixed parasites [3], 67 and 90-kDa lectin purified proteins [4, 5], synthetic peptides [6], recombinant proteins [7–14], and trypomastigote excreted/secreted antigen (TESA). Of these antigens, TESA proteins have shown particular promise in different assay formats (ELISA-TESA, Western blot) and in several different laboratories and geographic settings [15–19]. Unfortunately, the production of TESA antigen requires adequate cell culture facilities and other sophisticated infrastructure that are not always available to laboratories in resource-poor settings. Thus, in Latin American countries, diagnostic and research laboratories need practical, economical, and accurate tests that do not require special equipment [20]. Axenic growth of epimastigotes has long been used as a source of whole or purified *T. cruzi* antigens due to its simplicity and high antigen yields [15, 20–24]. To our knowledge, an ELISA based upon the excreted-secreted antigens of these epimastigote cultures has never been formally tested. The aim of the present study was to develop a simple, economical, and accurate ELISA using *T. cruzi* epimastigote secreted/excreted antigens (ELISA-ESEA) for the diagnosis of Chagas disease in areas with limited resources.

## 2. Methods

### 2.1. *T. cruzi* Strain

Epimastigote forms of *T. cruzi* MHOM/VE/08/AU isolate were obtained from an acute chagasic patient at the Tropical Medicine Center (Universidad de Oriente, Venezuela) and the RHO/VE/03/RG1 isolate was obtained from a vector (*Rhodnius prolixus*) captured in La Llanada de Cangua, Arismendi municipality, Sucre state, Venezuela. Both isolates were confirmed as *T. cruzi* by parasitological and molecular biology methods [25, 26]. The isolates were classified as TcI, widely distributed in Venezuela, and maintained on blood agar with monthly passages and adapted to axenic cultures.

### 2.2. Axenic Cultures *T. cruzi*

Epimastigotes of the *T. cruzi* (isolates MHOM/VE/08/AU and RHO/VE/03/RG1) were grown in Schneider's insect medium with L-glutamine (pH 6.9) (Sigma, St. Louis, MO, USA) supplemented with 0.6% calcium chloride and sterilized by filtration. After sterilization, heat-inactivated fetal bovine serum (FBS; Internegocios S.A. Buenos Aires, Argentina: final 10%) and penicillin/streptomycin (Sigma, St. Louis, MO, USA: final 1%) were added (complete media). Epimastigotes were collected during the logarithmic growth phase, as previously described [15].

### 2.3. ESEA Proteins

ESEA proteins from axenic cultures were harvested during logarithmic growth using a protocol adapted from the production of TESA antigens described by Berrizbeitia et al. [16]. Briefly, epimastigotes collected on the 7th day of growth were washed four times with 3 volumes of Schneider's insect medium without FBS (complete media) and centrifuged 2,000 *g *for 10 min at 4°C. The pellet was resuspended in 10 mL of complete media without FBS. After 4 days of incubation at 27°C, the epimastigotes were centrifuged at 2,800 *g* for 20 min and the supernatant (ESEA proteins) was harvested and filtered through a Millipore membrane (0.22 *μ*m; Millipore, Bradford, MA, USA). Proteins were used immediately or stored at −80°C. Protein content of the ESEA was quantified using the Bradford assay (BioRad, Hercules, CA, USA).

### 2.4. SDS-PAGE

ESEA proteins were evaluated by SDS-PAGE carried out on 0.8 mm thick slabs containing 10% polyacrylamide according to Laemmli [27]. Electrophoresis was performed for 2 h in Tris-glycine electrode buffer (25 mM Tris, 192 mM glycine, 0.1% SDS, pH 8.3) at a constant voltage of 100 V. Silver staining was employed for protein visualization. Molecular weight markers (BioRad, Hercules, CA, USA) were included in each run. 

### 2.5. Western Blot Analyses

Proteins separated by SDS-PAGE were transferred to nitrocellulose sheets (MFS, Pleasanton, CA, USA) according to Towbin et al. [28] using a minitank electroblotter (BioRad, Hercules, CA, USA). The transfer was performed for 1 h at 4°C in 25 mM Tris, 192 mM glycine, and 20% v/v methanol (pH 8.3) at a constant current of 0.25 A. Membranes were blocked overnight at 4°C with phosphate buffered saline (PBS) containing 5% skimmed milk (Parmalat) and 0.1% Tween 20 (blocking solution) and then incubated for 2 h with a pool of confirmed positive chagasic sera diluted 1 : 400 in the blocking solution. Following four 5-min washes of PBS containing 0.05% Tween 20 (PBST), the membranes were incubated for 2 h with the appropriate dilution of horseradish peroxidase (HRP)-conjugated goat antihuman IgG (Perkin Elmer Life Science, Boston, MA, USA) diluted in PBS containing 5% skimmed milk and 0.1% Tween 20. The membranes were then washed four times with PBST, and the immune complexes were revealed using diaminobenzidine (Sigma, St. Louis, MO, USA).

### 2.6. Serum Samples

A panel of 120 serum samples was used in this study. Fifty were obtained from Venezuelan subjects with Chagas disease, as confirmed by a battery of three different serological tests, including immunofluorescence, indirect hemagglutination, and ELISA, at the National Chagas Immunodiagnosis Laboratory (NCIL, Maracay, Venezuela). Samples were considered positive if the results of two out of the three assays were positive. The other 70 serum samples (controls) included 50 that were negative for all three serological tests and 20 from individuals with the following parasitic diseases: leishmaniasis (*n* = 10), ascariasis (*n* = 8), strongyloidiasis (*n* = 1), and trichuriasis (*n* = 1), obtained from the NCIL (Maracay, Venezuela) and the Diagnostic Laboratory for Infectious Diseases (Universidad de Oriente, Núcleo de Sucre, Venezuela).

### 2.7. ESEA-Based ELISAs

The coating of 96 well-polystyrene plates (Immulon 2; Thermo Labsystems, Franklin, MA, USA) was accomplished by incubating ESEA from the AU isolate (5,0 *μ*g/mL) at 4°C overnight (100 *μ*L/well) in 1 M sodium carbonate buffer (pH 9.6). The plates were then washed four times with PBS (pH 7.4; 0.01 M phosphate buffer, 0.14 M NaCl) containing 0.05% Tween 20 (A&C, American Chemicals Ltd., St-Laurent, QC, Canada) (PBST) and blocked for 1 h at 37°C with PBS containing 5% bovine serum albumin (Sigma, St. Louis, MO, USA) and 0.1% Tween 20. Sera were diluted 1 : 400 in the corresponding blocking solution 100 *μ*L/well and incubated for 1 h at 37°C. These dilutions were selected to permit optimal differentiation between positive and negative control sera, as tested by checkerboard titration (data not shown). Assays were completed with an optimal dilution of horseradish peroxidase-conjugated goat anti-human immunoglobulin G (IgG; 100 *μ*L/well; Perkin-Elmer Life Science, Boston, MA, USA) for 30 min at 37°C, four washes with PBST, and a final incubation with 3,3′, 5,5′-tetramethylbenzidine (100 *μ*L/well; Serologicals Corporation, Billerica, MA, USA) for 10 min at room temperature. The reaction was stopped with 1 N H_2_SO_4_ (50 *μ*L/well). Optical density (OD) was measured at 450 nm using an automated ELISA reader (Biotrak II, Amersham Biosciences, Cambridge, UK). All experiments were performed in duplicate on different days. Results were only accepted when the coefficient of variation within and among plates was <15%; otherwise, the samples were retested. The cut-off value for positivity was determined using a receiver-operating characteristic (ROC) curve analysis to establish the optimal sensitivity and specificity for the assay.

## 3. Results

### 3.1. ESEA Production

ESEA antigens were obtained from Schneider's insect medium supernatant on the fourth day of culture without purification procedures. The protein concentration in 60 mL of the axenic culture was 16.18 *μ*g/mL. The parasite density at the time of harvest was 1 × 10^7^ parasites/mL.

### 3.2. Identification of ESEA Proteins in the Supernatants of Axenic Cultures

**Figure 1:**
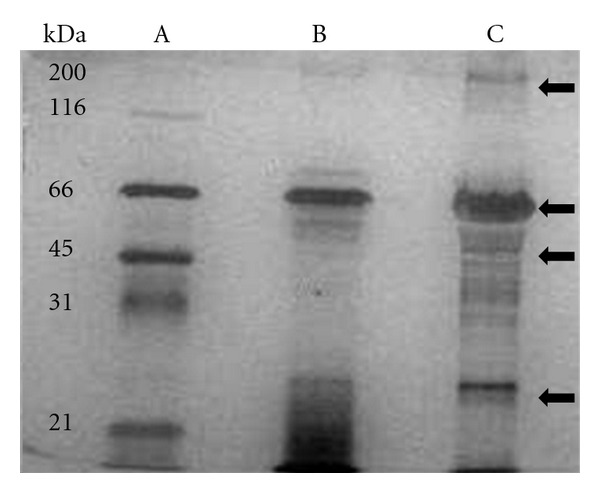
SDS-PAGE separation of the ESEA proteins visualized by silver staining. (A) (standard), (B) AU isolate, and (C) RG1 isolate. Arrows: common bands in both isolates.

**Figure 2:**
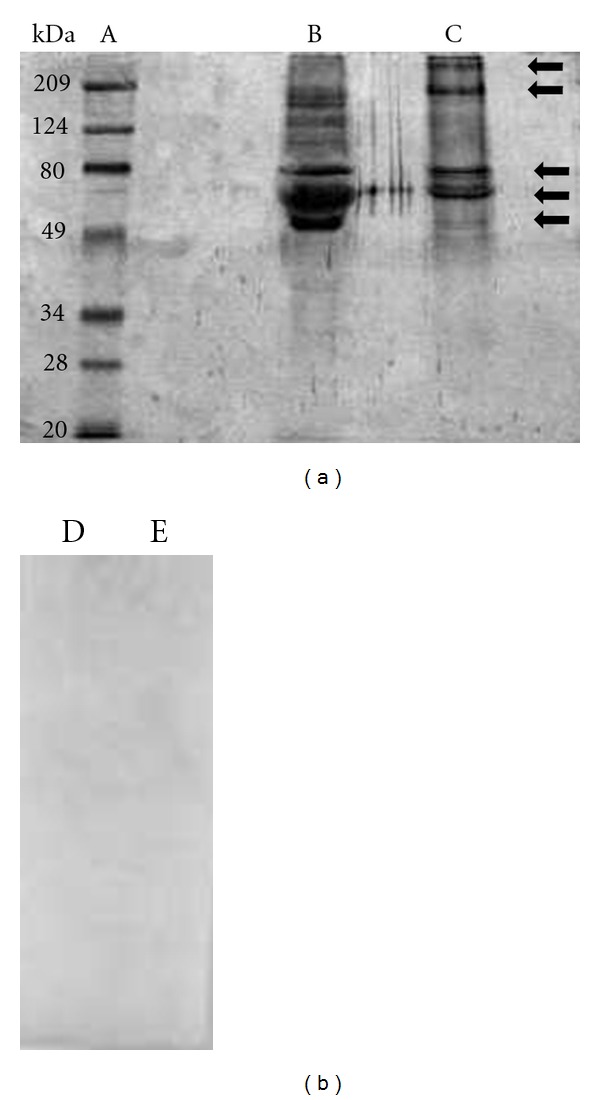
Western blot analysis of sodium dodecyl-polyacrylamide gel electrophoresis-separated ESEAs: Au and RG1 *T. cruzi* isolates. (A) standard, (B) RG1 isolate, and (C) AU isolate. The dot was probed with a pool of sera from patients with Chagas disease (B and C) and with a pool of control-negative sera RG1 and Au isolate, (D and E), respectively.

We performed SDS PAGE and WB analysis with pooled chagasic sera for the two isolates (MHOM/VE/08/AU and RHO/VE/03/RG1) to identify the immunoreactive polypeptide bands present in the supernatant of axenic *T. cruzi* cultures. The ESEA proteins, separated by SDS-PAGE and visualized with silver staining (Figure 1), varied both in number and molecular weights between the parasite isolates. The RG1 isolate had more protein bands than the AU isolate but there also appeared to be shared bands of approximately 25, 45, 66, and 200 kDa. Western blots also revealed the presence of immunogenic high molecular weight proteins, some of which were not visualized by silver stain of the SDS-PAGE. The pooled chagasic sera recognized ESEA polypeptides from both isolates, with molecular weights from 50 to over 200 kDa. Moreover, a pool of sera from healthy individuals did not react with the ESEA polypeptide bands (Figure 2). To our knowledge, this is the first paper that has both identified and showed the immunogenicity of ESEA proteins that could be used for the diagnosis of Chagas disease under different formats.

### 3.3. ELISA ESEA in Patient Samples

The ELISA ESEA was standardized using the MHOM/VE/08/AU isolate since this isolate had been obtained from an acute case of Chagas disease. Using checkerboard titrations, the best antigen concentration, as well as dilutions of the primary and secondary antibodies was determined to be 5 *μ*g/mL, 1/400, and 1/32000, respectively. After assay optimization, the mean optical density (OD) values for the pooled chagasic sera and negative control sera were 1.753 and 0.212, respectively (approximately 8 : 1). Generally, ELISA standardization requires this ratio to be at least 5 : 1 and a ratio of >10 is considered to be excellent [29].

**Figure 3:**
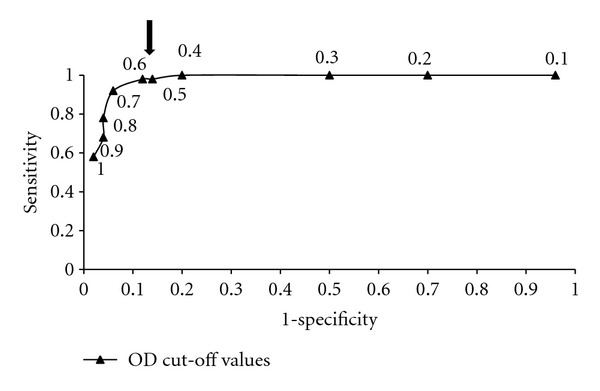
Receiver-operating characteristic curve for sensitivity and specificity (1-SP) in the enzyme-linked immunosorbent assays using ESEA antigens (AU isolate) at different arbitrary optical density (OD) cut-off values. Arrow: cutoff (0.600).

Forty-nine out of the 50 serum samples from subjects with confirmed Chagas disease were reactive in the ELISA ESEA when an OD cut-off value of 0.600 was used (sensitivity, 98%; 95% confidence interval, 96 to 100%) (Table 1). Six of the sera from healthy individuals (6/50) (12%), four from patients with leishmaniasis (4/10) (40%), three with ascariasis (3/8) (37.5%) and 1 with strongyloidiasis [100%] were reactive in this assay (specificity, 80%). The mean OD values for chagasic sera, the negative control sera, *Leishmania*-positive sera and sera, from other parasitosis were 1.099 ± 0.323 (range, 0.474 to 1.888), 0.339 ± 0.222 (range, 0.073 to 1.129), 0.617 ± 0.244 (range, 0.370–1.137), and 0.492 ± 0.275 (range, 0.250 to 0.921), respectively, (Table 2). Cut-off values between ODs of 0.40 to 0.60 yielded excellent sensitivity (98 to100%) (Table 2). The cut-off value 0.6 was determined after using a receiver operating curve (ROC) (Figure 3). This gave the best values for sensitivity (98%), specificity (88%), positive predictive value (PPV, 89%), and negative predictive value (NPV, 98%) (Table 2).

## 4. Discussion

To our knowledge, we are the first to describe the possible use of ESEA antigens for the diagnosis of Chagas disease. ESEA antigens are simple and inexpensive to produce and protein yields can be very high. In addition, these antigens show an excellent sensitivity and an acceptable specificity in the ELISA format. In this study, we evaluated the use of ESEA antigens that could feasibly be produced in laboratories with scarce financial resources. For this reason, we adapted the protocol for the production of TESA antigens that have previously been shown to be excellent for the diagnosis of Chagas disease, in order to produce ESEA antigens [16]. Although TESA antigens represent an excellent diagnostic alternative for *T. cruzi *infections, they must be produced in cell cultures and thus require expensive laboratory equipment and infrastructure. This tends to increase the cost of producing TESA antigens, thus limiting their use in Latin America where resources are scarce. One of the most important advantages of ESEA antigens in relation to epimastigote-based ELISAs is that ESEA antigens are very stable and do not need protease inhibitors. Furthermore, reactivity is retained for approximately one year after production. Another advantage of ESEA antigens compared to other antigenic preparations from epimastigotes is the easy production since they donot need to be sonicated or follow other biochemical procedures to obtain usable antigenic preparations [20, 30, 31].

The yield obtained for ESEA antigens was high enough to be usefully applied in diagnostic serological tests, giving an adequate quantity of ESEA antigens for diagnosis even with a low production of epimastigotes. Umezawa et al. [18] reported a similar concentration of excreted/secreted proteins in the supernatant of Vero cells (30–40 *μ*g/mL) and demonstrated their usefulness for the diagnosis of Chagas disease.

In this study we demonstrated that ESEA antigens are a mix of polypeptide bands ranging from 20 to 220 kDa that are secreted/excreted by epimastigote forms into the supernantant of axenic media. These ESEA antigens are immunogenic and the sera taken from the chagasic patients recognized these protein bands in a range from 50 to 220 kDa. To our knowledge, this is the first study that demonstrates the immunoreactivity of ESEA proteins. Moreover, using just two isolates, we were able to demonstrate that the bands vary in number and relative molecular weight. Kesper Jr. et al. [17] also demonstrated a unique banding pattern that is characteristic for each strain or isolate in *T. cruzi* trypomastigote exoantigens.

After demonstrating the detection of ESEA antigens by a pool of chagasic sera in the Western blot format, we evaluated the usefulness of these proteins for the diagnosis of Chagas disease in the ELISA format. The ESEA-ELISA using the AU strain was 98% sensitive and 88% specific, with the latter value being due to cross-reactions with sera from patients with leishmaniasis and other parasitosis. These results are similar to those of other investigations. Cannnova et al. [32] standardized an ELISA assay using a crude epimastigote antigen; the sensitivity and specificity of the test was 96% and 97%, respectively. Moreover, Telles et al. [33] used *T. cruzi* ubiquitin as a differential diagnostic antigen to distinguish tripanosomiasis and leshmaniosis with an ELISA test at a sensitivity and specificity of 98% and 93.8%, respectively, Additionally, Schechter et al. [34] developed an ELISA assay using a 90 kDa purified protein, the sensibility and specificity of this assay are 96.6% and 91.9%, respectively.

One of the most important aspects for the practicality and usefulness of diagnostic tests is the type of antigen to be used; its antigenic performance and the simplicity of its production that result in a practical method and avoid laborious and difficult procedures: ESEA antigens have all of these characteristics.

TESA antigens have been used widely for the detection of Chagas disease in different formats (Western blot, ELISA, multiple antigen binding assay: MABA) and show excellent sensitivity and specificity for all of these diagnostic assays [16, 19, 35, 36]. Additionally, these antigens are used in Brazilian blood banks for confirmatory testing or to classify inconclusive results of *T. cruzi* infections [37]. Nevertheless, TESA production is both complicated and expensive. Thus, ESEA antigens provide an attractive alternative strategy for the diagnosis of this parasitic disease since these antigens are easier and cheaper to produce. Epimastigotes grow easily and rapidly in broth media, and high yields are obtained after the logarithmic growth phase.

Although ESEA-ELISA showed very high sensitivity (98%), the test had limitations as regards the specificity: six sera from healthy patients, four from patients with leishmaniasis, and four with other parasitic diseases gave false positive reactions. As has been reported previously, limitations associated with specificity in the diagnosis of Chagas disease have been reported by other authors, and patients infected with *Leishmania* spp. are the most likely to cross-react [16, 19, 36]. In order to correct for this error, positive ELISA-ESEA samples should be confirmed by a second serological and more specific assay such as Western blotting.

In addition to confirming the potential efficacy of the ESEA antigens for the diagnosis of *T. cruzi* infections, we also demonstrated their advantages. Large amounts of ESEA antigens were obtained using a simple and inexpensive protocol (six 25 cm^2^ flasks yielded approximately 60 mL of ESEA proteins at 16.18 *μ*g/mL). The yield from six flasks would permit the coating of approximately 21 ELISA plates and the performance of 851 tests.

In summary, we believe that although TESA antigens have a superior performance in terms of sensitivity and specificity, our ELISA-ESEA antigens have several advantages. These include efficiency, ease of production, and high yields that ensure sufficient assays can be undertaken. Moreover, our study is the first to describe the immunogenic bands in the ESEA antigens that are recognized by chagasic sera. A study validation of ELISA-ESEA in the field is clearly needed but these antigens certainly represent an alternative for Chagas disease diagnosis in laboratories with scarce resources.

## Figures and Tables

**Table 1:** Sensitivity, specificity, positive predictive values, and negative predictive values of the ESEA-ELISA at various arbitrary OD cut-off values.

OD cut-off Values and parameters	ESEA-based assay
0.400	
Specificity (%) (95% CI)	74 (62–86)
Sensitivity (%) (95% CI)	100
NPV (%) (95% CI)	100
PPV (%) (95% CI)	79 (69–89)

0.500	
Specificity (%) (95% CI)	80 (69–91)
Sensitivity (%) (95% CI)	98 (94–100)
NPV (%) (95% CI)	98 (93–100)
PPV (%) (95% CI)	83 (73–93)

0.600	
Specificity (%) (95% CI)	88 (79–97)
Sensitivity (%) (95% CI)	98 (96–100)
NPV (%) (95% CI)	98 (94–100)
PPV (%) (95% CI)	89 (81–97)

NPV: negative predictive value.

PPV: positive predictive value.

CI: confidence interval.

**Table 2:** Mean, standard deviation (SD), optical density range (OD), and specificity estimates for serum groups in ELISAs based on secreted and excreted antigen homogenates (ESEA antigens) from trypomastigote forms of *T. cruzi*.

Group of individuals	Mean (OD) ± SD	Range DO min–max	Sp % (DO 0,500)	Sens % (DO 0,500)	Sp % (DO 0,600)	Sens % (DO 0,600)
Individuals negative for *T. cruzi* infection (*n* = 50)	0.339 ± 0.222	0.073–1.129	80	—	88	—
Chagas-positive individuals (*n* = 50)	1.099 ± 0.323	0.474–1.888	—	98	—	98
*Leishmaniasis*-positive individuals (*n* = 10)	0.617 ± 0.244	0.370–1.137	30	—	60	—
Individuals-positive for other parasitic diseases (*n* = 10)	0.492 ± 0.275	0.205–0.921	50	—	60	—

OD: optical density; SD: standard deviation; min: minimum, max: maximum; Sens: sensitivity; Sp: specificity.
